# Expanding the Biological Properties of Alkannins and Shikonins: Their Impact on Adipogenesis and Life Expectancy in Nematodes

**DOI:** 10.3389/fphar.2022.909285

**Published:** 2022-06-08

**Authors:** Athanasios S. Arampatzis, Olga Tsave, Benjamin Kirchweger, Julia Zwirchmayr, Vassilios P. Papageorgiou, Judith M. Rollinger, Andreana N. Assimopoulou

**Affiliations:** ^1^ Organic Chemistry Laboratory, School of Chemical Engineering, Aristotle University of Thessaloniki and Natural Products Research Centre of Excellence (NatPro-AUTH), Center for Interdisciplinary Research and Innovation of AUTh (CIRI-AUTh), Thessaloniki, Greece; ^2^ Division of Pharmacognosy, Department of Pharmaceutical Sciences, Faculty of Life Sciences, University of Vienna, Vienna, Austria

**Keywords:** alkannin, shikonin, hydroxynaphthoquinones, structure activity relationship, 3T3-L1 adipogenesis, *Caenorhabditis elegans*, nematotoxicity, chiral natural products

## Abstract

Alkannin, shikonin (A/S) and their derivatives are naturally occurring hydroxynaphthoquinones biosynthesized in some species of the Boraginaceae family. These natural compounds have been extensively investigated for their biological properties over the last 40 years, demonstrating a plethora of activities, such as wound healing, regenerative, anti-inflammatory, antitumor, antimicrobial and antioxidant. This study aims to extend the current knowledge by investigating the effects of various A/S compounds on two model systems, namely on 3T3-L1 pre-adipocytes and the nematode *Caenorhabditis elegans*. The former constitutes an established *in vitro* model for investigating anti-obesity and insulin-mimetic properties, while the latter has been widely used as a model organism for studying fat accumulation, lifespan and the anthelmintic potential. A set of chemically well-defined A/S derivatives were screened for their effect on pre-adipocytes to assess cell toxicity, cell morphology, and cell differentiation. The differentiation of pre-adipocytes into mature adipocytes was examined upon treatment with A/S compounds in the presence/absence of insulin, aiming to establish a structure-activity relationship. The majority of A/S compounds induced cell proliferation at sub-micromolar concentrations. The ester derivatives exhibited higher IC_50_ values, and thus, proved to be less toxic to 3T3-L1 cells. The parent molecules, A and S tested at 1 μM resulted in a truncated differentiation with a reduced number of forming lipids, whereas compounds lacking the side chain hydroxyl group projected higher populations of mature adipocytes. In *C. elegans* mutant strain SS104, A/S enriched extracts were not able to inhibit the fat accumulation but resulted in a drastic shortage of survival. Thus, the set of A/S compounds were tested at 15 and 60 μg/ml in the wild-type strain N2 for their nematocidal activity, which is of relevance for the discovery of anthelmintic drugs. The most pronounced nematocidal activity was observed for naphthazarin and β,β-dimethyl-acryl-shikonin, followed by isovaleryl-shikonin. The latter 2 A/S esters were identified as the most abundant constituents in the mixture of A/S derivatives isolated from *Alkanna tinctoria* (L.) Tausch. Taken together, the findings show that the structural variations in the moiety of A/S compounds significantly impact the modulation of their biological activities in both model systems investigated in this study.

## 1 Introduction

Alkannin and Shikonin (A/S) are naturally occurring naphthoquinones that comprise—together with their derivatives [acetyl-shikonin (ACS), isovaleryl-shikonin (IVS), deoxy-shikonin (DS), and β,β-dimethyl-acryl-shikonin (DMAS); Table 1]—the main active components of the roots of several medicinal plants, belonging to the Boraginaceae family (Papageorgiou et al., 1999). These small molecules have attracted the attention of numerous research groups, due to their remarkable biological potential. More specifically, a series of pharmacological properties have been attributed to A/S and their derivatives over the years, with the anti-inflammatory, antimicrobial, anticancer, antioxidant, as well as wound healing and regenerative effects being the most important ones. More importantly, several pharmaceutical preparations invented by Prof. Papageorgiou of our group have been approved by the National Organization of Medicines in Greece, for their strong wound healing activity, proved by multiple clinical trials (Papageorgiou et al., 2008). Added to that, a number of *in vitro* and *in vivo* studies published the last 15 years have reported the beneficial effects of S and its derivatives on metabolic diseases, such as diabetes mellitus (DM) (Papageorgiou et al., 1999; Papageorgiou et al., 2008; Andújar et al., 2013; Guo et al., 2019).

**TABLE 1:** Chemical structures of A/S derivatives tested.

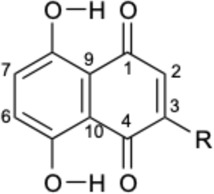					
**R**	**Name**	**MW**	**R**	**Name**	**MW**
H	Naphthazarin (NAPH)	190.15	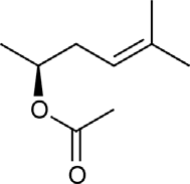	Acetyl-shikonin (ACS)	330.30
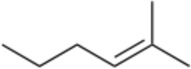	Deoxy-shikonin (DS)	272.29	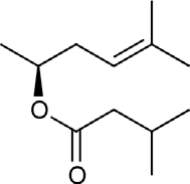	Isovaleryl-shikonin (IVS)	372.40
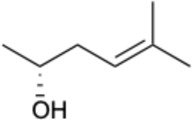	Alkannin (A)	288.29	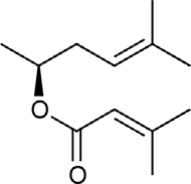	β,β-Dimethyl-acryl-shikonin (DMAS)	370.40
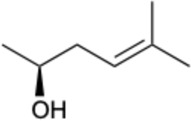	Shikonin (S)	288.29			

DM is defined as a group of heterogeneous metabolic disorders with chronic hyperglycemia as the common characteristic end point phenotype in all cases (Forouhi and Wareham, 2014). Hyperglycemia emerges following a series of pathological processes related (in)directly to the failure to produce or respond to insulin (Brown and Walker, 2016). DM and obesity have an intimate relationship, with both being closely associated with adipose tissue. Its accumulation during obesity is linked to tolerance observed toward insulin (Yazıcı and Sezer, 2017). Given that the disease has a rapid rise in prevalence worldwide, along with the unmet therapeutic needs, an immediate need for new insulin-mimetic pharmaceuticals arises. A significant number of studies deal with the discovery and optimization of novel insulin mimetic agents with increased insulin-mimetic capacity (Kuzulugil et al., 2019).

*Caenorhabditis elegans* (*C. elegans*) and other invertebrate models are increasingly utilized in chemical screens to identify compounds that inhibit fat accumulation and increase lifespan (Lemieux et al., 2011; Ackerman and Gems, 2012; Shen et al., 2018). The worms are 1 mm in size, robust and easily maintained in the laboratory on nematode growth medium (NGM) agar plates. Its simplicity and small size enable sample testing at the scale of conventional cellular assays and further requires only small amounts of test samples (O’Reilly et al., 2014). Triglyceride stores can be easily quantified *via* biochemical, chromatographic or dye-based methods (e.g., Nile red, oil red O) (Lemieux and Ashrafi, 2015). Hence, *in vivo* sample screens are feasible which would be impossible with vertebrate models. Compared with well-established rodent models of obesity and diabetes *C. elegans* screening has the advantages of: i) a higher throughput of bulk populations, ii) resource-saving testing due to the small size of the worm. Therefore, test substances are only required in μg-mg quantities, iii) few laboratory safety measures and legal regulations on the use of *C. elegans* (Harvey-Clark, 2011; Hunt, 2017), as well as iv) a suite of genetic tools available to elaborate on findings (Singh, 2021). Of course, these advantages also come at the expense of translatability to human pathologies (Kleinert et al., 2018). However, it is acknowledged that many regulators of human fat storage, utilization and longevity have orthologues in the nematodes e.g., DAF-2, the worm orthologue of the human insulin receptor (35% sequence identity) regulates glucose transporters, autophagy, lipid metabolism and lifespan of the worm (Kimura et al., 1997; Murphy, 2013). Recently, a 96-well based screening platform to measure both, survival and fat accumulation in *C. elegans* was established by some of the co-authors (Zwirchmayr et al., 2020). However, apart from the objective to discover compounds able to reduce fat accumulation and increase the lifespan of *C. elegans*, the nematodes have emerged as an important pre-screening model system for anthelmintic drug discovery. The complex life cycle of human parasites belonging to the nematode phylum poses a great challenge for a fast and efficient compound screening. Hereby, *C. elegans* is a convenient and proficient surrogate. It was shown that compounds nematocidal to *C. elegans* are 15 times more likely to be nematocidal to other parasitic nematodes than randomly selected compounds (Burns et al., 2015).

Various studies have been performed during the last 2 decades, attempting to shed light on the effect of S and its derivatives on obesity and obesity-associated diseases, like DM. S has been found to impede differentiation of 3T3-L1 pre-adipocytes, through downregulation of various adipogenesis-related factors, such as peroxisome proliferator-activated receptor γ (PPAR-γ), CCAAT/enhancer binding protein α (C/EBPα), sterol regulatory element binding protein 1C (SREBP1C), KROX20 and Kruppel-like factor 15 (KLF15) (Lee H.-Y. et al., 2009; Lee et al., 2010). Added to that, different molecular mechanisms seem to be involved in the anti-adipogenic activity of S, as for example the modulation of Wnt/β-catenin pathway (Lee et al., 2010), the inhibition of extracellular signal-regulated kinases 1 and 2 (ERK1/2) phosphorylation (Gwon et al., 2013) and the downregulation of microRNA (miRNA)/FK506 binding protein 1B (FKBP1B) pathway (Jang et al., 2015). S was also shown to possess insulin-like properties (Nigorikawa et al., 2006) and stimulate glucose uptake *in vitro* (Kamei et al., 2002) and *in vivo* (Öberg et al., 2011). Moreover, *in vivo* S-treatment resulted in decreased weight gain and hepatic fat accumulation, enhanced glucose tolerance, augmented fatty acid oxidation-associated genes and ameliorated hepatic insulin signaling in mice (Bettaieb et al., 2015; Gwon et al., 2015). The anti-diabetic potential of S has been further demonstrated in recent studies, which showed that S could act as a free fatty acid receptor 4 (FFA4) agonist in HT-29 cells and lower plasma glucose levels in diabetic mice (Xu et al., 2021), whereas it suppressed protein tyrosine phosphate 1B (PTP1B), during *in silico* and *in vitro* experiments (Saeed et al., 2021). At this point, it should be noted that, to the best of our knowledge, there has not been a related study on the effect of either the A enantiomer or the chirality of A/S on DM/obesity/glucose uptake.

Another key point that needs to be stressed is that most of the commercial S samples used in the above studies were not characterized in terms of identity, purity and chirality. The characterization step is of utmost importance, especially in biological experiments, where activities can be attributed to compounds that may not completely correspond to the correctly identified enantiomer and/or pure compound; hence, biological activity has been reported to be affected by chirality (Nguyen et al., 2006). This mischaracterization of commercial A/S samples was reported by our group in previous studies, showing that several commercial samples were misnamed by the suppliers with respect to their chirality as A or S (Tappeiner et al., 2014).

Similar to the above, various A/S derivatives have exhibited anti-obesity and anti-diabetic activities, such as a commercial sample of ACS (Huang et al., 2019) isolated from *Lithospermum erythrorhizon* Siebold and Zucc. root extracts (Gwon et al., 2012; Su M. L. et al., 2016; Su M et al., 2016), DMAS from *Arnebia euchroma* (Royle) I. M. Johnst. ethanol extract (Pandeti et al., 2016), as well as a commercial sample of IVS (Ha et al., 2016). Finally, the extracts from different parts of boraginaceous plants (*L. erythrorhizon* Siebold and Zucc. roots, *Onosma hispida* Wall. ex G. Don roots, *O. dichroantha* Boss. shoots, leaves and roots, and *A. euchroma* (Royle) I. M. Johnst. leaves) have been documented to exhibit beneficial effects on regulating blood glucose levels in animal models (Kumar et al., 2010; Ko et al., 2013; Naderi et al., 2017; Noorafshan et al., 2017).

Although there has been a substantial amount of research on the anti-adipogenic/anti-obesity effect of hydroxy-naphthoquinones and their extracts, most of the publications focus on S and a few other derivatives. In this regard, our study aimed at exploring the impact of several A/S derivatives (fully identified) on adipogenesis under a structure-activity relationship (SAR) approach. More specifically, a series of A/S derivatives were assessed against 3T3-L1 pre-adipocytes for: i) their cell toxicity at different concentrations after 24 and 48 h, ii) their effect on adipocyte differentiation at different concentrations, and iii) their insulin-mimetic action, by fully substituting insulin during the differentiation process. The tested compounds were selected with the purpose to identify the pharmacophore moiety of the various hydoxynaphthoquinone structures and study the effects of the various substitution patterns. In this regard, based on the bioactivity of A and S, we started from their parent molecule, naphthazarin (NAPH), followed by assessing the impact of the isohexenyl side-chain addition (corresponding to deoxy-shikonin; DS), as well as the substitution of the side chain hydrogen with a chiral hydroxyl group (representing S or A). Furthermore, to examine the effect of the chirality, we included in our analysis both of the enantiomers A and S, as well as a mixture of A and S with an intermediate chirality (enantiomeric ratio S:A 42:58%). Finally, different acyl-substituted A/S derivatives were further studied to explore the activity of compounds that induce steric hindrance phenomena and increased lipophilicity and eccentricity. To further evaluate the effect of A/S samples *in vivo*, a hexane extract from the roots of *A. tinctoria* (L.) Tausch and an A/S-enriched fraction thereof were tested in a *C*. *elegans* fat accumulation model (Zwirchmayr et al., 2020). Nematocidal activity of these samples was shown in a *C*. *elegans* survival assay.

## 2 Materials and Methods

### 2.1 Chemicals and Reagents

For the 3T3-L1 experiments, purified and commercially purchased A/S compounds (Table 1) were tested, including monomeric S (S predominates, S:A% ratio 77.7:22.3), monomeric A (A predominates, A:S% ratio 89.3:10.7), a mixture of S and A (42:58% enantiomeric ratio by chiral-HPLC analysis) [S-A (42:58)], NAPH, DS, acetyl-shikonin (ACS), isovaleryl-shikonin (IVS) and β,β-dimethyl-acryl-shikonin (DMAS). Monomeric S was purified by A. Assimopoulou from a S commercial sample (Bioshikonin; Ichimaru Pharcos Co., Ltd.) by silica gel column chromatography. Its purity and identity were determined by HPLC (purity >98%), chiral LC and LC-MS. Monomeric A was isolated and purified by A. Assimopoulou from a commercial sample (Ikeda Corp.) by Sephadex LH-20 column chromatography (dichloromethane and acetone eluants). Its purity and identity were determined by HPLC (purity >98%), chiral LC and LC-DAD-MS. Purified NAPH was obtained by recrystallization with n-hexane from a NAPH commercial sample (Fluka, Buchs, Switzerland, LOT: 000475-38-7). Commercial samples of DS (TCI Europe, Lot AV21-VQ), ACS (ABCR GmbH and Co.), IVS (TCI Europe, Lot AV41-UP) and DMAS (ABCR GmbH and Co., Lot AV11-XP) were examined for their identity and purity by HPLC-DAD. In addition, an hexane extract (HE) from *A. tinctoria* (L.) Tausch roots (Soft-N-Supple, Pakistan) was tested, along with an A/S mixture (A/S-mix) that was isolated from the above hexane extract, based on the protocol proposed by Prof. Papageorgiou (Assimopoulou et al., 2009) and was analyzed for A/S derivatives by HPLC-DAD (Agilent Technologies, Germany). The A/S-mix consists of isovaleryl-A/S 37.4%, β,β-dimethyl-acryl-A/S 37.24%, acetyl-A/S 20.43%, DS 4.01%, A/S 0.78%, and propionyl-A/S (PS) 0.14% w/w.

Dulbecco’s Modified Eagle’s Medium (DMEM), fetal bovine serum (FBS), penicillin/streptomycin, trypsin-EDTA, 3-Isobutyl-methyl-xanthine (IBMX), dexamethasone (DEX), human recombinant insulin and Oil Red O were obtained from Sigma-Aldrich (United States). CellTiter-Glo luminescent cell viability assay was purchased from Promega Corporation (Madison, WI, United States). Dimethyl sulfoxide (DMSO), formaldehyde 3.7%–4.0% w/v and TRItidy-G were obtained from AppliChem (Germany). iScript cDNA synthesis kit and iTaq Universal SYBR Green Supermix were purchased from Bio-Rad laboratories (United States).

Regarding the experiments in the *C. elegans* fat accumulation assay, HE and A/S-mix from *A. tinctoria* (L.) Tausch roots were used (2.5, 10 and 25 μg/ml). Whereas in the survival assay only the A/S mixture together with the purified A/S compounds [S, A, S-A (42:58), NAPH, DS, IVS, and DMAS] were assayed.

### 2.2 *In vitro* Fat Accumulation Model (3T3-L1 Pre-Adipocytes)

#### 2.2.1 Cell Culture and Differentiation

Mouse fibroblast 3T3-L1 preadipocytes were kindly donated by the Laboratory of Inorganic Chemistry, School of Chemical Engineering, Aristotle University of Thessaloniki (Thessaloniki, Greece) and cultured in 75 cm^2^ cell culture flasks in high-glucose DMEM supplemented with 10% FBS, 100 units/mL penicillin and 100 ng/ml streptomycin at 37°C in 5% CO_2_ and standard humidity. Medium was changed three times per week, until cells reached confluence. Two days post-confluency (day 0), cells were induced to differentiate by treating them with a differentiation/induction medium containing DMEM supplemented with 0.5 mM IBMX, 1 μM DEX, 1 ng/ml of insulin, and 10% FBS. Three days later (day 3), the medium was replaced with DMEM containing 10% FBS and 1 μg/ml insulin for two more days, after which (day 5) the medium was changed to DMEM with 10% FBS. The medium was replaced every 2 days until cells were harvested on day 11 (Tsave et al., 2018).

#### 2.2.2 Cell Viability Assay

Viability of undifferentiated 3T3-L1 cells upon treatment with various concentrations of the hydroxynaphthoquinones was assessed using CellTiter-Glo assay in opaque-walled 96-well plates. This method utilizes the luciferase reaction for quantifying adenosine triphosphate (ATP), which is an indicator of cellular metabolic activity. Through the luciferase reaction, luminescence is generated in proportion to the amount of available ATP. Therefore, viable (metabolically active) cells produce a luminescent signal, whereas non-viable cells do not (Riss et al., 2004). Briefly, cells were seeded in a 96-well plate at a density of 3.5 × 10^3^ cells and a total volume of 100 μl per well. After 24 h incubation, cells were treated with different concentrations (10 nM–10 μΜ) of the hydroxynaphthoquinones for 24 and 48 h. After the respective incubation times, cells in each condition were treated with a volume of the reagent equal to the volume of cell culture medium present in each well. The luminescence was recorded with a Glomax 96 microplate luminometer (Promega Corporation, United States). Untreated cells served as control samples. Each assay was performed three times, with at least three replicates each.

All tested compounds were previously dissolved in DMSO. The final concentration of DMSO in each well was lower than 0.1%. To confirm that DMSO at that concentration was inert, a screening was performed at various concentrations (0.00002%–2%) and cell viability was assessed. Cell viability was expressed as the percentage of viability observed in each compound, compared to the untreated cells. The cell viability data were used for calculating the IC_50_ values after 24 h, by plotting the former (*y*-axis) against the log concentrations of the compounds (*x*-axis) in Microsoft Excel.

#### 2.2.3 Induction of Adipogenesis With Hydroxynaphthoquinones

3T3-L1 pre-adipocytes were differentiated into mature adipocytes following the aforementioned differentiation protocol (vide infra). Briefly, pre-adipocytes were treated with either 10 ng/ml of insulin and/or hydroxynaphthoquinones (100 nM or 1 μΜ). The tested compounds were first dissolved in DMSO and subsequently in the culture medium, with the final concentration of DMSO not exceeding 0.1% v/v. The initial stock concentration was 50 mM. Cells treated with insulin were considered as positive control whereas untreated cells were included as vehicle control. On the 11th day of the differentiation process, cell differentiation was assessed and validated by oil red O staining, as described elsewhere (Tsave et al., 2016). Further validation of successful adipogenesis was also confirmed by the relative expression of closely related biomarkers PPAR-γ and adiponectin (ADIPOQ).

#### 2.2.4 Oil Red O Staining

3T3-L1 preadipocytes were seeded in 24-well plates at a density of 3.5 × 10^4^ cells per well and differentiated as described above. On the 11th day, cells were washed three times with sterile PBS, fixed with formaldehyde 3.7%–4.0% for 20 min and stained with Oil Red O (ORO) solution for 10 min. Next, cells were washed with double distilled water (ddH_2_O) and stained lipids were observed and imaged under an inverted microscope, as described elsewhere (Tsave et al., 2016). The analysis and quantification of lipid accumulation—based on the amount of ORO dye—was performed by using ImageJ software (National Institutes of Health, United States). In brief, the RGB images with the stained lipids were converted to 8-bit grayscale (*Image* → *Type* → *8-bit*). Next, a manual threshold was set (*Image* → *Adjust* → *Threshold*) for each sample and the stained areas were measured (*Analyze* → *Measure*) (Mehlem et al., 2013).

#### 2.2.5 Reverse Transcription-Polymerase Chain Reaction Assay

Total RNA was extracted from cells on the 11th day of the differentiation protocol, using TRItidy-G reagent. The concentration and purity (absorbance ratio 260/280) of the extracted RNA were measured with a NanoDrop™ 2000c spectrophotometer (Thermo Fisher Scientific, United States). Synthesis of cDNA was performed with the iScript cDNA synthesis kit according to manufacturer instructions. RT-PCR was run on Rotor Gene Q (Qiagen) using the iTaq Universal SYBR Green Supermix and appropriate reagents (Qiagen, United States).

Customized primers were used from Qiagen for adiponectin (Mm_Adipoq_1_SG, NM_009605, Q01048047), GAPDH (Mm Gapdh_3_SG, QT01658692) and mPPAR-γ forward 5′-GTC​AGC​GAC​TGG​GAC​TTT​TC-3′ and reverse 5′-CGA​GGA​CAT​CCA​AGA​CAA​CC-3’.

### 2.3 *Caenorhabditis elegans* Models

#### 2.3.1 *Caenorhabditis elegans* Strain, Maintenance, and Synchronization

*Caenorhabditis elegans* wild-type var. Bristol N2, SS104 [glp-4 (bn2ts)] and *Escherichia coli* OP50 were provided by the *Caenorhabditis* Genetics Center (University of Minnesota, United States). Media and NGM agar plates have been prepared as described before (Zwirchmayr et al., 2020). OP50 were grown in LB medium for 8 h at 37°C, then harvested by centrifugation, washed twice with ddH_2_O and suspended in S-complete medium at 100 mg/ml. Worms were maintained on NGM agar plates inoculated with OP50. Synchronized cultures were prepared by egg-prep method (Porta-de-la-Riva et al., 2012) and experiments started at the L4 stage.

#### 2.3.2 Nile Red Assay

Nile red assay was performed as reported previously (Zwirchmayr et al., 2020). S-Medium, OP50 (10 mg/ml), Nile red (100 nM) and DMSO stock solutions were added to each well of a clear 96-well plate. Final concentration of DMSO was 1%. In each well 2–10 synchronized L4 SS104 nematodes were placed to reach a final volume of 100 μl per well. Test substances and vehicle control were tested in six well replicates. Worms were incubated in darkness at 25°C for 4 days. On day 4, worms were paralyzed for image acquisition by adding NaN_3_. For quantification of Nile red staining, fluorescence images of all living nematodes were acquired with a Zeiss Axio Observer Z1 with rhodamine filter using the same sub-saturating exposure time and settings. The open source software ImageJ was used for image processing and quantification of fluorescence as described recently (Lehner et al., 2021). Presented results are the mean worm fluorescence calculated from three independent experiments. Fluorescence is expressed as % of control worms ±SD, whereby control worm fluorescence is set to 100% fluorescence.

#### 2.3.3 Survival Assay

The survival assay was performed as described before: Briefly, a suspension of 5–18 synchronized L1 larvae (N2 wild-type) in S-complete medium were manually seeded into 96-well plates. OP50 at a concentration of 6 mg/ml was added immediately and worms were kept at 25°C. The following day, when all worms reached the L3 stage, the sterilizing-agent 5-Fluorodeoxyuridine (FUdR; 0.12 mM final; Sigma-Aldrich, F0503) was added to keep the population synchronized. The next day, samples were added in triplicate wells to the adult worm culture with DMSO as a carrier at a final concentration of 1%. The number of living worms in each well was counted on day 0, 3, 5, 7 and 10 of the treatment. The worms were oxygenized every 3 days and OP50 were added on day 5 of adulthood to prevent starvation. Results of three parallel experiments are presented as bar charts and are given as the mean survival rate of worms.

### 2.4 Statistical Analysis

The data are presented as mean value ±standard deviation (SD). One-way analysis of variance (ANOVA) was performed for 3T3-L1 cell viability assays and Nile red staining, followed by Tukey’s and Dunnett’s post-hoc multiple comparison tests, respectively. Differences with *p*-value (p*) < 0.05 were considered statistically significant. All statistical analyses were performed using SPSS 25.0.

## 3 Results

### 3.1 *In vitro* Fat Accumulation Model (3T3-L1 Pre-Adipocytes)

#### 3.1.1 Cytotoxicity of A/S and Derivatives Towards 3T3-L1 Cells

**FIGURE 1:**
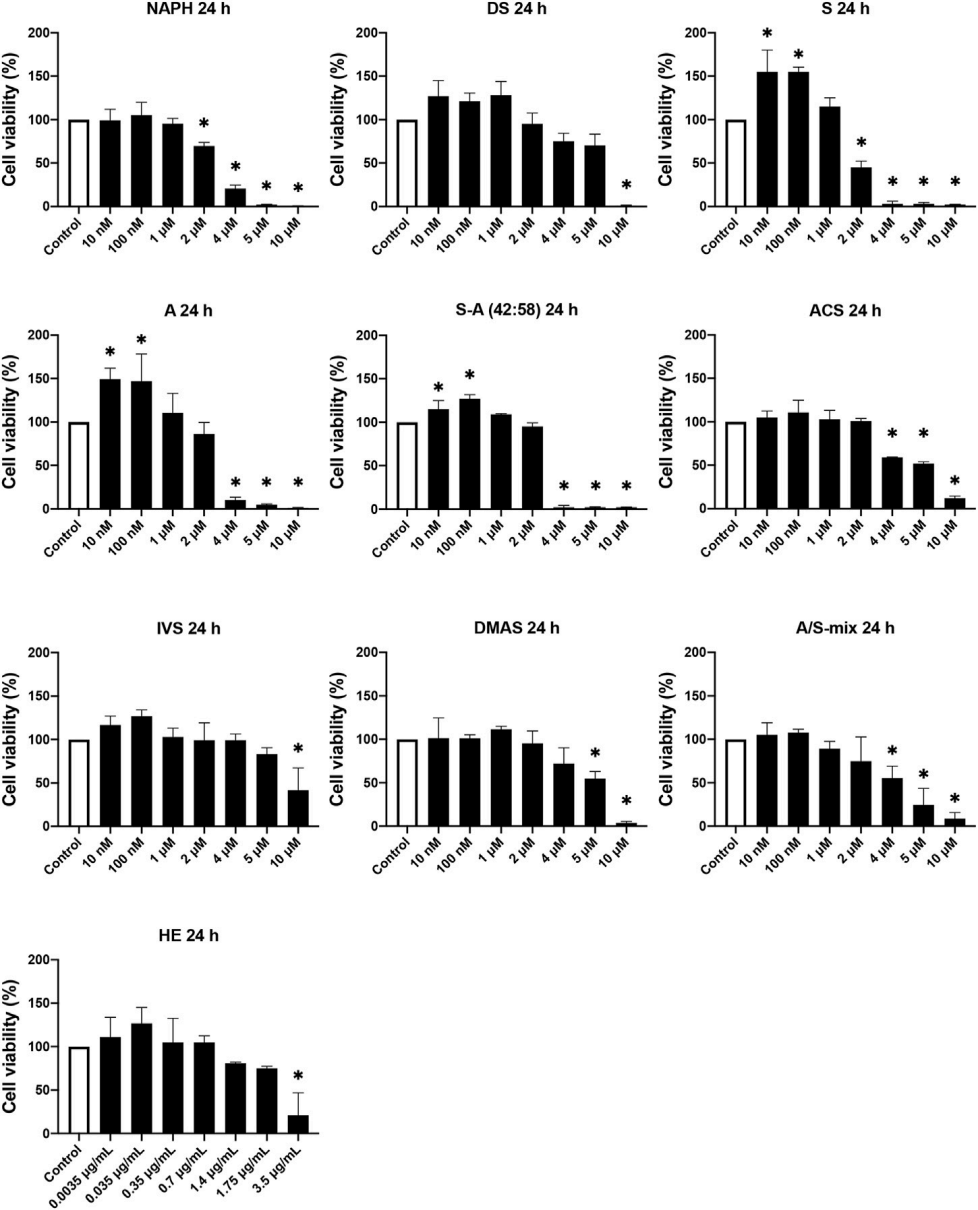
Cell viability of 3T3-L1 pre-adipocyte cells upon treatment with various A/S derivatives at 24 h * indicates *p* < 0.05 compared to untreated (control) cells. Cell viability values are expressed as the percentage of viability observed in untreated cells (100% cell viability). Data are shown as the mean of three individual experiments ±SD.

To study the effect of A/S compounds on the differentiation of 3T3-L1 pre-adipocytes, first it was important to examine the cytotoxic profile of A/S and derivatives to ensure that no toxicity is being observed upon exposure of cells to the compounds for more than 24 h. Therefore, HE and A/S-mix (containing mainly isovaleryl-, β,β-dimethyl-acryl-, acetyl-, deoxy-, propionyl-A/S, and A/S), followed by NAPH, DS, S, A, S-A (42:58), ACS, IVS, and DMAS, were tested at various concentrations (10 nM–10 μM for pure compounds or A/S-mix and 0.0035–3.5 μg/ml for HE) for 24 and 48 h. Figure 1 shows that all samples were found non-toxic for concentrations up to 1 μM, during the 24 h incubation. In fact, the A/S esters (ACS, IVS, DMAS), as well as DS, HE, and A/S-mix samples showed reduced cell viability percentages at even higher concentrations (>4 μM or >0.7 μg/ml for HE). Yet, this was not the case for NAPH, S, A, and S-A (42:58), which exhibited significantly decreased cell viability percentages at >4 μM, e.g., the two enantiomers, S and A, at the concentration of 4 μM inhibited cell viability by more than 90%. Furthermore, it was interesting that S, A, and S-A (42:58) managed to elicit a significant cell proliferation for relatively low concentrations (0.01, 0.1, and 1 μM), with the former two exhibiting cell viability values of higher than 146%. Overall, differences between the A, S and S-A (42:58) samples were observed, with most of them not being statistically significant. Only at 2 μM these differences were more pronounced and statistically significant, with A exhibiting less toxicity than S.

**FIGURE 2:**
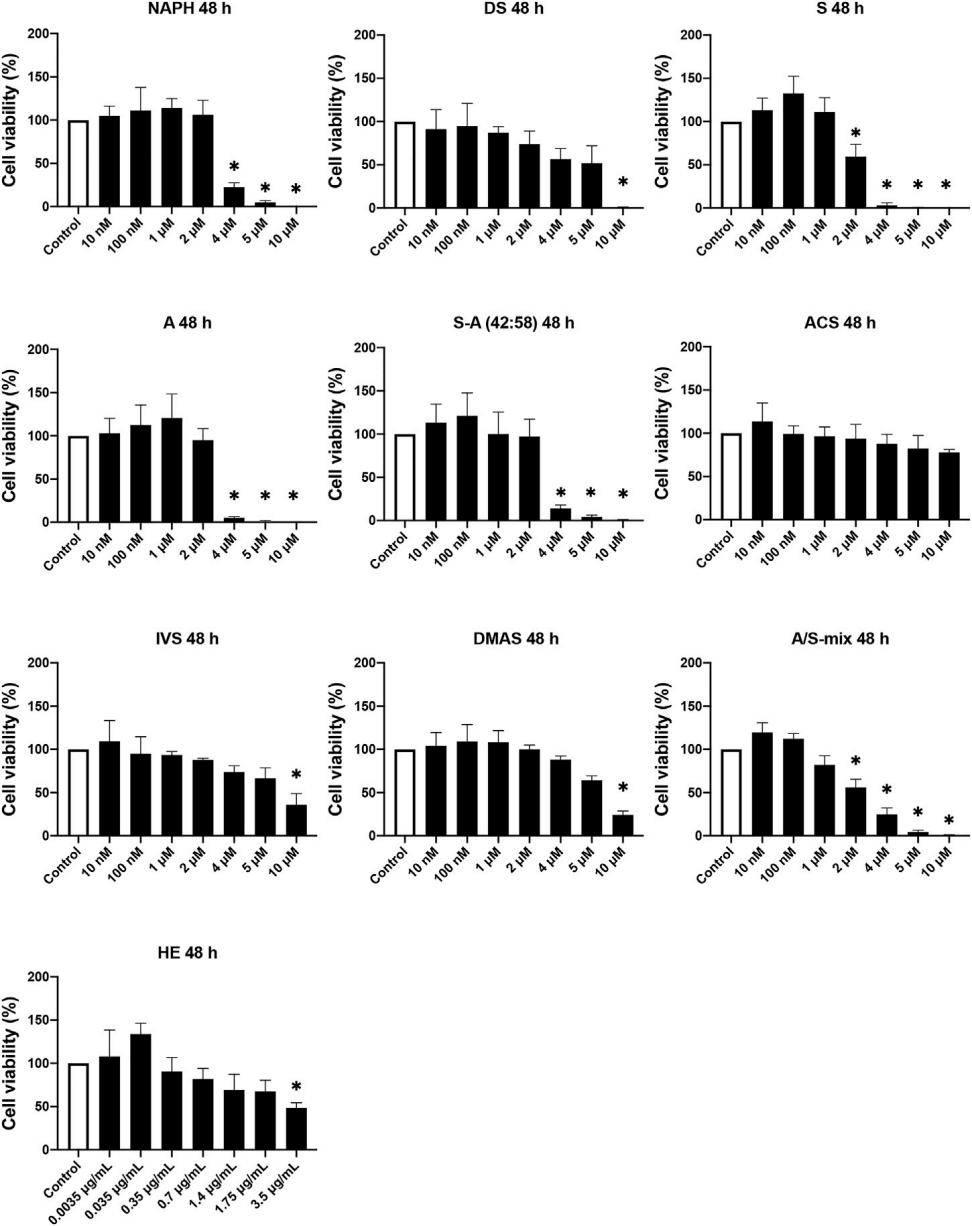
Cell viability of 3T3-L1 pre-adipocyte cells upon treatment with various A/S derivatives at 48 h * indicates *p* < 0.05 compared to untreated (control) cells. Cell viability values are expressed as the percentage of viability observed in untreated cells (100% cell viability). Data are shown as the mean of three individual experiments ±SD.

Regarding 48 h cell viability assay, Figure 2 shows that most compounds exhibited a toxic profile similar to the corresponding one in the 24 h assay. For concentrations up to 1 μM at 48 h, DS, S, and A showed considerable decreases in cell viability compared to 24 h, however they were not significant. On the contrary, the toxic effects induced by NAPH, ACS, and DMAS at the higher (2–10 μM) concentration range, after 48 h, were reversed. Surprisingly, for cells that were treated with A/S-mix at 2–10 μM, there was a reduction in viability (compared to 24 h) ranging from 46% (2 μM) to almost 100% (10 μM). By contrast, at lower concentrations (10 and 100 nM), A/S-mix induced cell proliferation, increasing cell viability percentages higher than 100%. Last, the profile of S-A (42:58), IVS, and HE samples remained almost unchanged, in comparison to 24 h.

**FIGURE 3:**
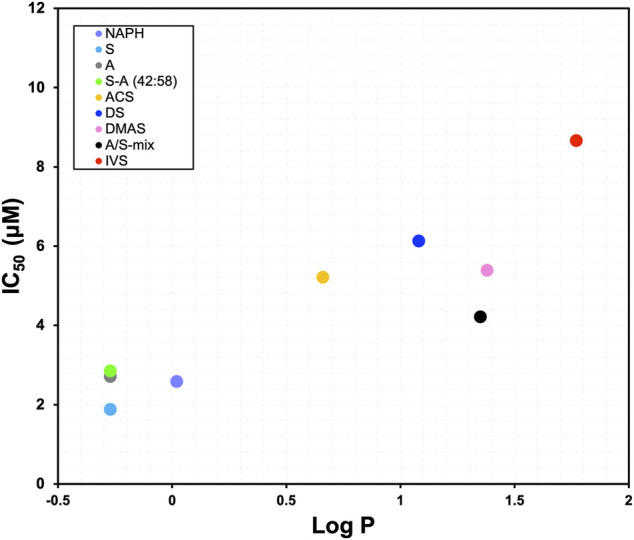
Relationship between IC_50_ values after 24 h and Log *p* values for the A/S derivatives [NAPH, DS, S, A, S-A (42:58), DMAS]. A/S compounds are shown in order of increasing hydrophobicity; e.g., S more hydrophilic, IVS more hydrophobic. Log *p* values were calculated through ChemDraw 20.0 Professional free trial (PerkinElmer Informatics Inc., 1985-2020) software, as in Ordoudi et al. (2011).

From the 24 h cell viability data we further proceeded to calculate the IC_50_ values for each derivative and plotted them against computed Log *p* values. The scatterplot depicted in Figure 3 demonstrates the relationship between different structures of A/S compounds and their cytotoxic activities. It can be noticed that A/S derivatives with higher Log *p* values (more hydrophobic) are correlated with higher IC_50_ values, e.g., IVS with a Log *p* of 1.77 displayed an IC_50_ value of 8.66 μM. By contrast, the less hydrophobic NAPH (Log *p* 0.02) and the enantiomers S (Log *p* −0.27) and A (Log *p* −0.27), together with their mixture S-A (42:58; Log *p* −0.27)) exhibited lower (<3 μM) IC_50_ values.

#### 3.1.2 Evaluation of Adipogenesis of 3T3-L1 Cells in the Presence of A/S and Derivatives by ORO Stain

For the assessment of adipogenesis, the following derivatives were selected: NAPH, DS, S, A, S-A (42:58) and DMAS, aiming to investigate the effect of the presence of the isohexenyl-chain to the NAPH moiety, further addition of the hydroxyl group, the impact of A/S enantiomeric ratio, as well as the influence of hydroxyl group esterification.

To investigate the effect of A/S and derivatives on adipogenesis, we induced the differentiation of 3T3-L1 preadipocytes into mature adipocytes according to a standard protocol (Tsave et al., 2018). Cells were subjected to differentiation with the selected A/S compounds (at 100 nM and 1 μM) in the presence of insulin. This allowed us to examine the potential inhibitory/inducive activity of the hydroxynaphthoquinones. The lipids of differentiated 3T3-L1 cells were stained by using ORO.

**FIGURE 4:**
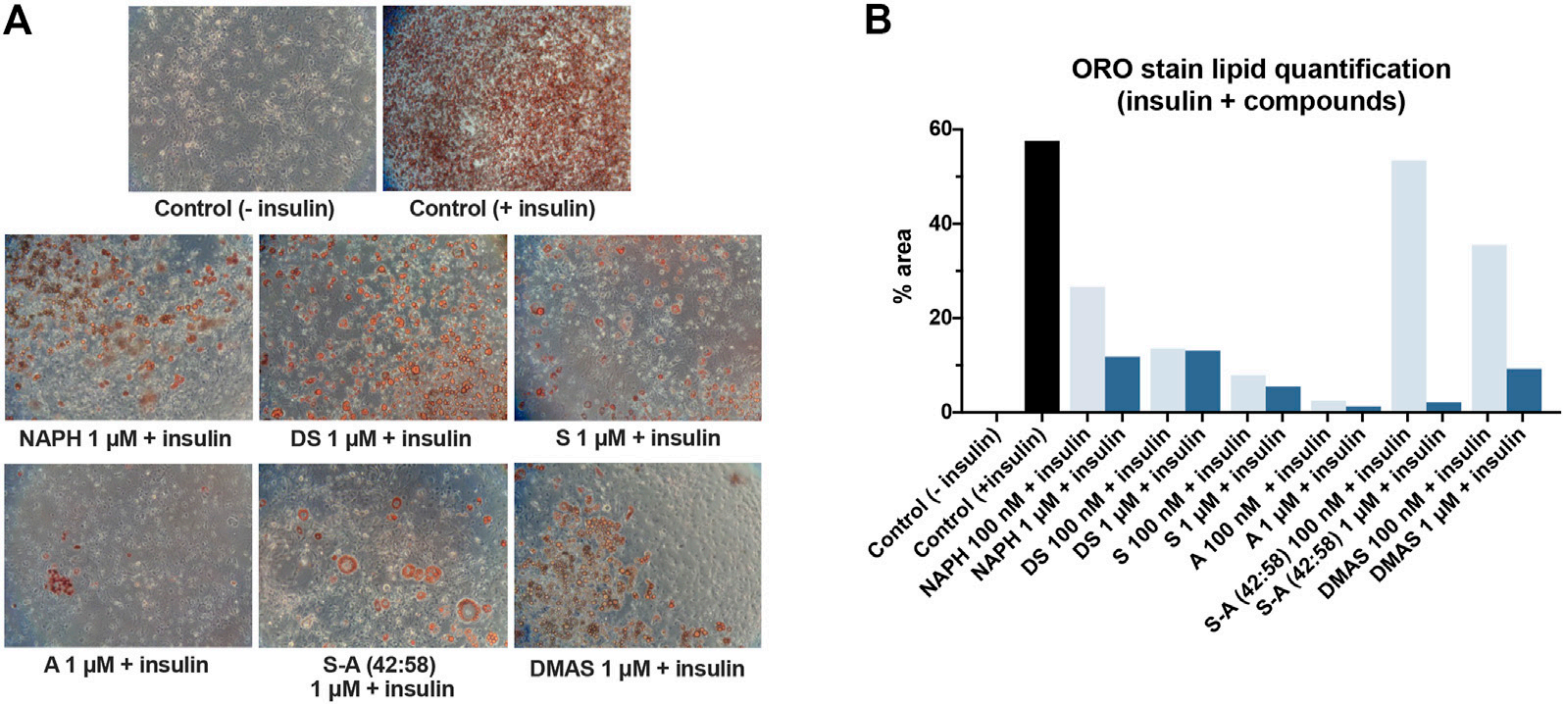
Differentiation of 3T3-L1 cells in the presence of insulin and A/S derivatives. **(A)** Representative microscopic images of the stained cells for the various samples at 1 μM. All images were recorded at ×10 magnification. Control (−insulin) and control (+insulin) represent negative and positive control samples, respectively. **(B)** Lipid quantification based on the amount of ORO stain present in each sample.

As shown in Figure 4, treatment of cells with any of the tested compounds in the presence of insulin led to a compromised lipid formation as opposed to the positive control sample, where the lipid-containing differentiated cells covered the entire surface. It can also be noticed from the microscopic images in Figure 4A that the parent molecules, S and A, together with their mixture 42:58, caused the highest adipogenic inhibition; at a concentration of 1 μM they showed a 22-fold, 37-fold, and 27-fold decrease in lipid accumulation, respectively (Figure 4B). In the case of DMAS the formed lipids accounted for 9.24%, while the corresponding percentages of NAPH and DS exceeded 10%. Figure 4B also highlights that all A/S compounds exerted their activity in a concentration-dependent fashion, with 100 nM resulting in a reduced adipogenic inhibition. This difference was more pronounced mainly for S-A (42:58), DMAS and NAPH.

Next, we were prompted to test the insulin-like activity of A/S and their derivatives, thus we induced the differentiation of 3T3-L1 cells by fully substituting insulin with the A/S compounds (100 nM and 1 μM). As previously, cell differentiation was assessed through ORO staining.

**FIGURE 5:**
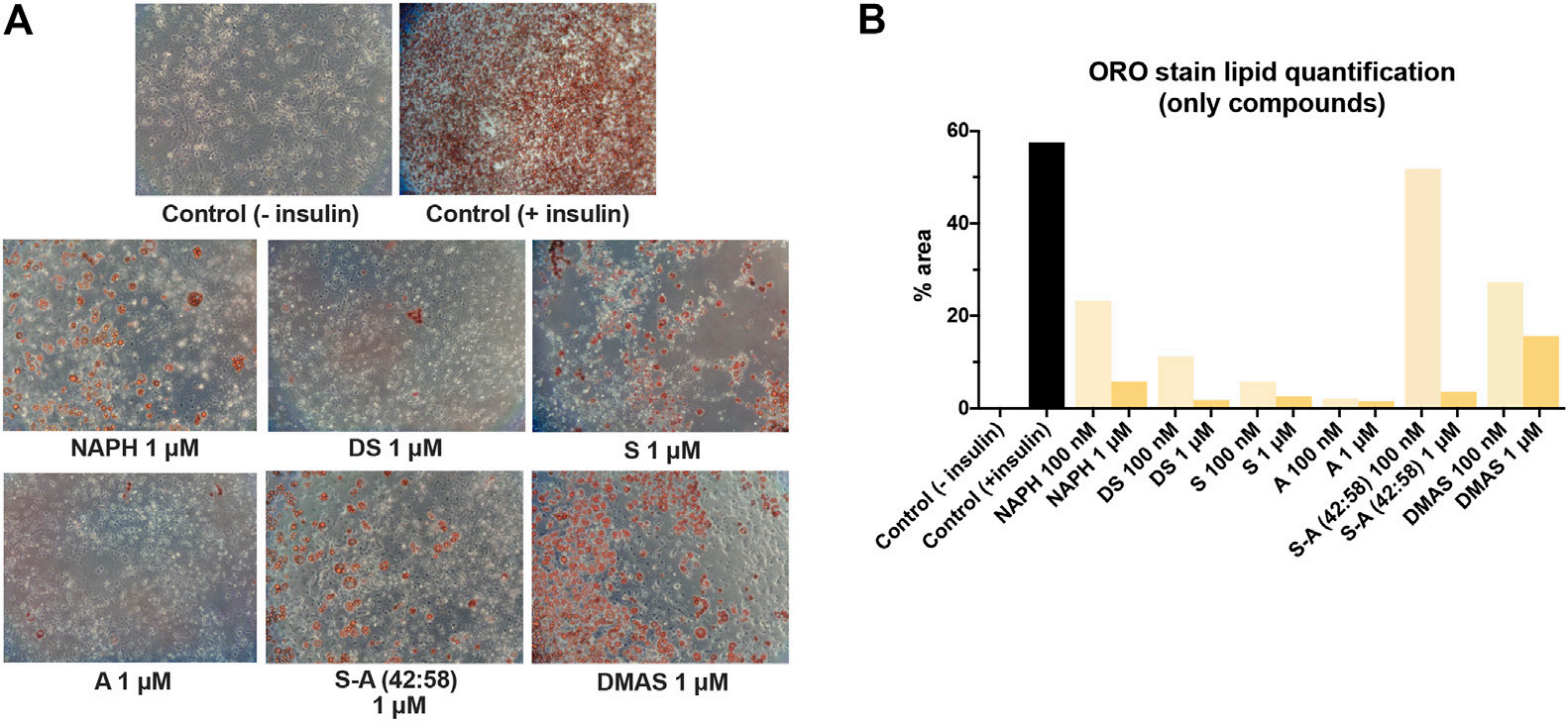
Differentiation of 3T3-L1 cells in the presence only of A/S derivatives. **(A)** Representative microscopic images of the stained cells for the various samples at 1 μM. Control (−insulin) and control (+insulin) represent negative and positive control samples, respectively. All images were recorded at ×10 magnification. **(B)** Lipid quantification based on the amount of ORO stain present in each sample.

In Figure 5, lipid formation was attenuated to different extents upon treatment with 1 μM of hydroxynaphthoquinones, when compared to positive control (insulin-induced) samples. A and DS caused strong inhibition of adipogenesis (1.56% and 1.78% lipid accumulation, respectively), followed by S (2.58%), S-A (42:58) (3.16%), and NAPH (5.74%). On the contrary, DMAS displayed a lipid accumulation of 15.65%; thus, inducing lipid formation (compared to negative control sample), yet attenuated (compared to positive control sample). Cells treated with 100 nM of A/S and derivatives in the absence of insulin (Figure 5B) produced comparable results to those observed previously, in compound-treated cells under the presence of insulin; lipid accumulation was augmented to varying degrees (2–51.8%). Once more, S-A (42:58), DMAS, and then NAPH, showed the highest increments, with the first compound reaching approximately 90% of the lipid amount present in the positive control sample.

**FIGURE 6:**
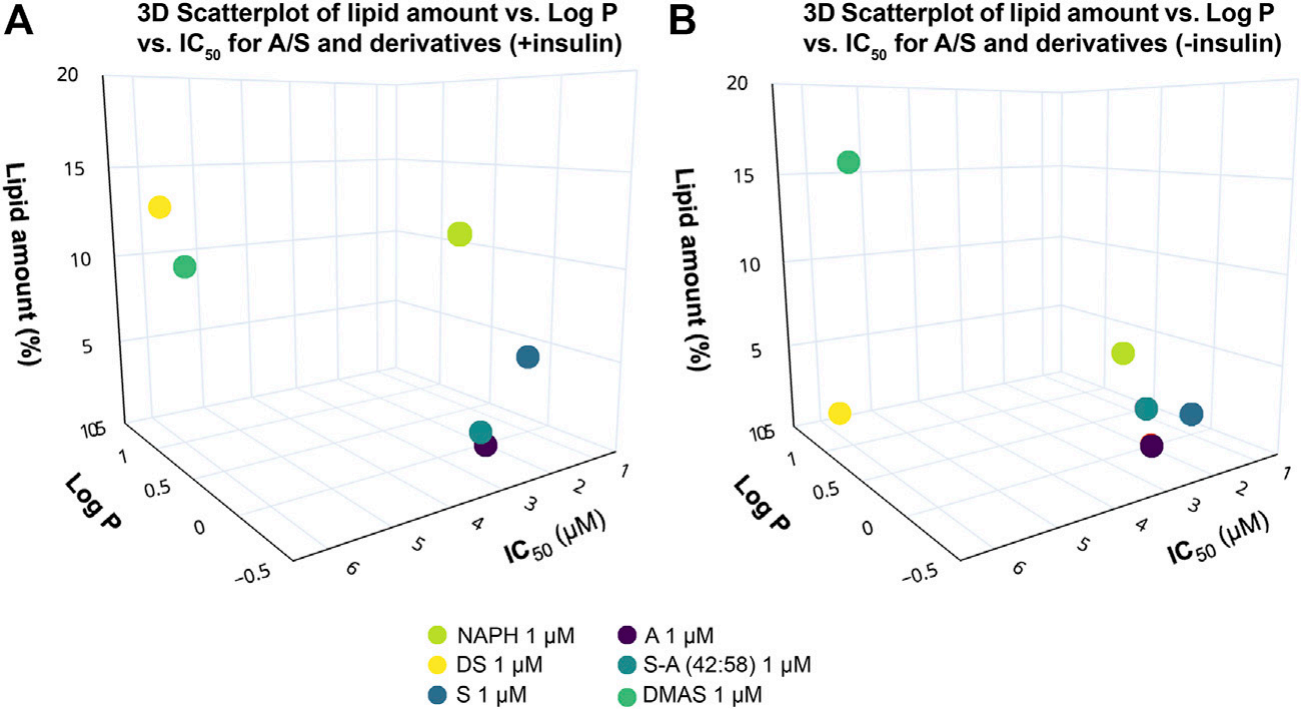
3D scatterplots showing the correlation between lipid amount in 3T3-L1 cells treated with A/S and derivatives [NAPH, DS, S-A (42:58), DMAS; 1 μM], Log *p* and IC_50_ values of the A/S compounds in the **(A)** presence and **(B)** absence of insulin. Compounds bearing the OH group in the side chain [S, A and S-A (42:58)] exhibited a stronger inhibition of lipid formation, in the absence of insulin, enhancing the anti-adipogenic activity. The 3D scatterplots were generated using Plot. ly Chart Studio (available at https://chartstudio.plot.ly; Plotly Technologies Inc., 2022).

To gain a better overview of the data from the ORO assay, we correlated them with the Log *p* and IC_50_ values of the hydroxynaphthoquinones by constructing 3D scatterplots. Figure 6A illustrates that the two enantiomers (S and A) and their 42:58 enantiomeric mixture, demonstrating similar IC_50_ values, had a stronger inhibitory effect on adipogenesis in contrast to the rest of compounds. The more hydrophobic compounds (DS and DMAS), as well as the more hydrophilic NAPH—all lacking the side-chain hydroxyl group—showed increased lipid accumulation percentages. Based on the above observations, it is apparent that the presence of hydroxyl group in the side chain of A/S plays an important role on lipid inhibition. In the case where no insulin was administered (Figure 6B), S, A, and S-A (42:58) preserved their suppressive activity, while NAPH and DS had also a suppressive effect on lipid accumulation. As it was discussed above, DMAS elicited an even weaker inhibition against 3T3-L1 differentiation.

#### 3.1.3 Effect of A/S and Their Derivatives on the mRNA Expression of PPAR-γ and ADIPOQ

In addition to the ORO assay, we wanted to assess the differentiation of 3T3-L1 cells—upon treatment with A/S and their derivatives—by measuring the relative mRNA expression of selected molecular targets that are closely associated with adipogenesis. In this respect, we studied the expression of PPAR-γ and ADIPOQ; the former acts as a transcription factor in the early stages of differentiation process and determines the successful progression of adipogenesis, whereas the latter is highly expressed in mature adipocytes. As previously mentioned, 3T3-L1 cells were induced to differentiate in the presence of A/S compounds together with insulin or the compounds alone. On the 11th day, total mRNA was isolated and RT-PCR analysis was performed for quantifying PPAR-γ and ADIPOQ expression.

**FIGURE 7:**
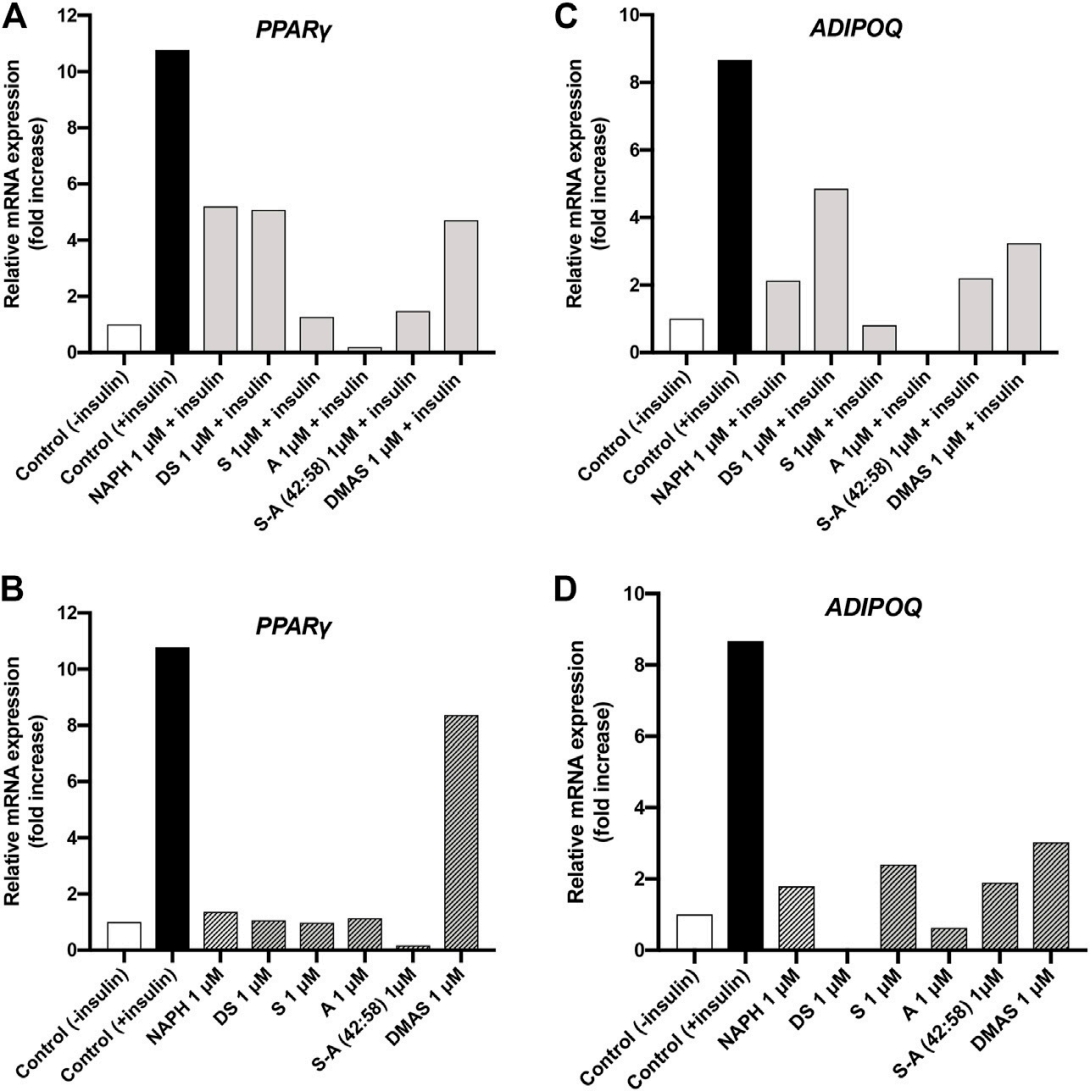
Relative concentration of mRNA expression for PPAR-γ and ADIPOQ in differentiated 3T3-L1 cells treated with A/S and their derivatives in the presence **(A,C)** and absence of insulin **(B,D)**.

Figure 7A illustrates that the expression of PPAR-γ in all samples where insulin and A/S compounds were present, remained lower compared to the positive control sample (10.78-fold increase). Yet, different responses were observed among the tested hydroxynaphthoquinones; S, A, and S-A (42:58) exhibited 1.27-, 0.20-, and 1.48-fold increases, respectively, while for NAPH, DS, and DMAS the corresponding increases were 5.2-, 5.08-, and 4.71-fold, respectively. When 3T3-L1 cells were induced to differentiate with the A/S compounds alone (Figure 7B), all samples showed an attenuated PPAR-γ expression (0.16–1.36-fold increase) except for DMAS, which demonstrated an 8.37-fold increase. Nevertheless, the expression of PPAR-γ was markedly reduced for all samples when compared to the insulin group (positive control).

Results from the analysis of mRNA expression of ADIPOQ indicated that treatment of 3T3-L1 cells with A/S and their derivatives—in the presence (Figure 7C) or absence (Figure 7D) of insulin—led to the expression of ADIPOQ, yet lower compared to positive control sample (8.67-fold increase). Specifically, S and A (Figure 7C) exhibited the strongest inhibitory activities against ADIPOQ expression, with the former showing a smaller increase than negative control sample (1-fold increase) and the latter not showing any increase at all. Furthermore, NAPH and S-A (42:58) demonstrated fold increases of 2.13 and 2.2, respectively, followed by DMAS (3.24-fold increase) and DS (4.85-fold increase). Upon treatment with the hydroxynaphthoquinones alone (Figure 7D), the attenuated expression of ADIPOQ was retained, with DS and A showing the strongest inhibition. S-A (42:58), S, and NAPH demonstrated comparable fold increases (1.89, 2.4 and 1.79, respectively), while DMAS displayed a higher fold increase (3.03).

### 3.2 Studies in *Caenorhabditis elegans*

**FIGURE 8:**
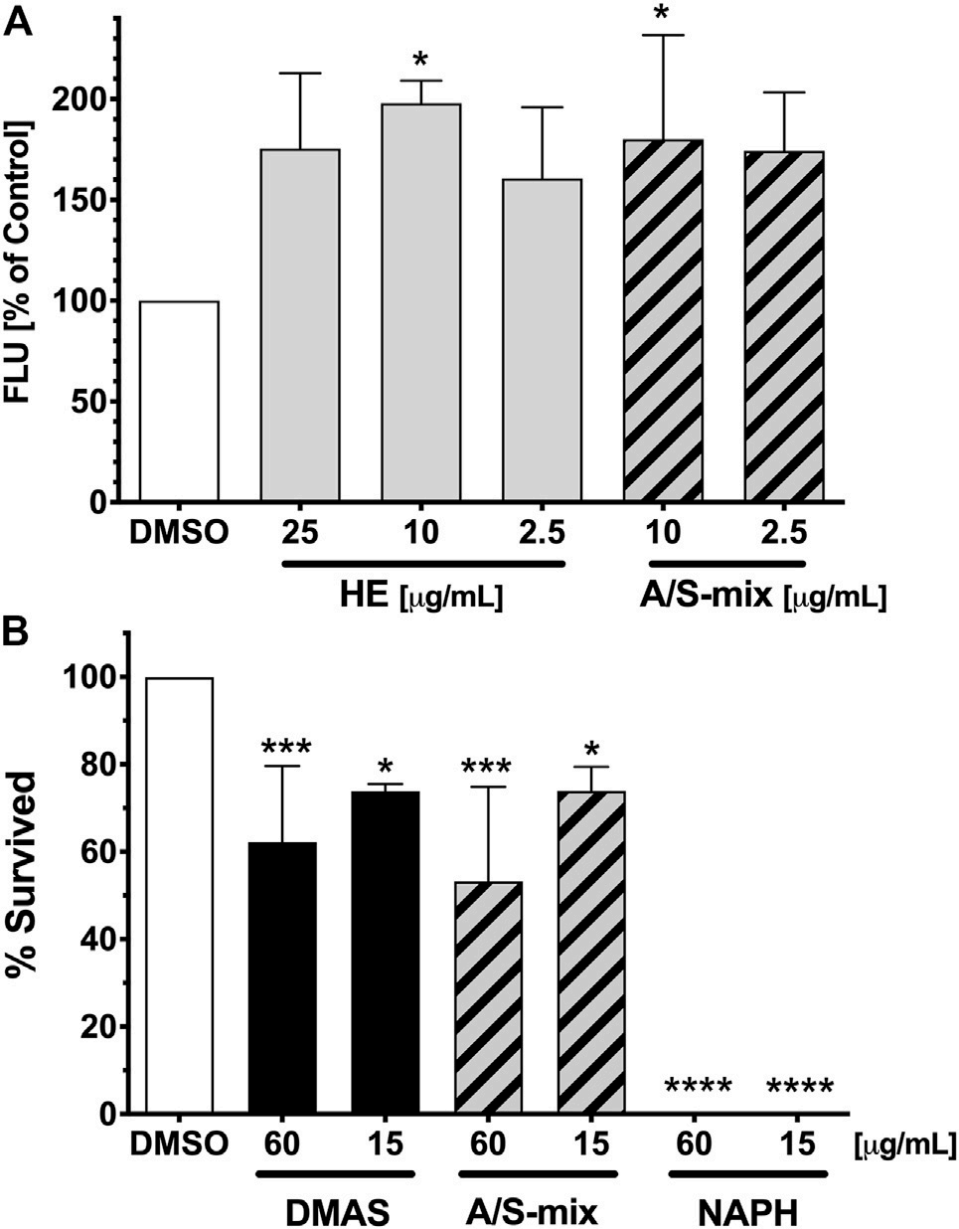
Fat Accumulation and survival of *C. elegans* upon treatment with *Alkanna tinctoria* (L.) Tausch samples. **(A)** Differences in vital Nile red fluorescence (FLU) of *C. elegans* treated with vehicle control (DMSO 1%), and different concentrations of HE and A/S-mix. Bars represent the mean fluorescence of three independent experiments with six well replicates expressed as % of control worms ±SD. The presented values were obtained by measuring the fluorescence of 44–71 worms per sample. Significance was assessed by One-Way ANOVA and Dunnett’s post-test (*, *p* < 0.05). **(B)** Survival rates (%) after 10 days of treatment with the vehicle control (DMSO 1%) and the samples DMAS, A/S-mix and NAPH. All sample were tested at a concentration of 60 and 15 μg/ml. Bars represent the mean survival rate of three independent experiments ±SD. The presented values were obtained by assaying a total of 67–93 worms per sample. Significance was assessed by One-Way ANOVA and Dunnett’s post-test (*, *p* < 0.05; ***, *p* < 0.001; ****, *p* < 0.0001).

To further explore the effects of *Alkanna tinctoria* (L.) Tausch *in vivo*, HE as well as the A/S-mix were tested in a *C. elegans* fat accumulation model based on Nile red lipid staining (Figure 8A). Interestingly, HE showed an increase of lipid derived Nile red fluorescence at all tested concentrations (2.5, 10, and 25 μg/ml). At 10 μg/ml, the fluorescence of HE treated worms was nearly twice as high (197.8% ± 11.19%) as the vehicle treated worm fluorescence. Similar effects were observed for the A/S-mix at 10 and 2.5 μg/ml with a worm fluorescence of 180.2% (±51.52) and 174.3% (±28.95) of vehicle treated worms. Higher concentrations could not be tested, because worms treated with 25 and 100 μg/ml of the A/S-mix were found dead or with an abnormal morphology after 4 days of treatment.

**FIGURE 9:**
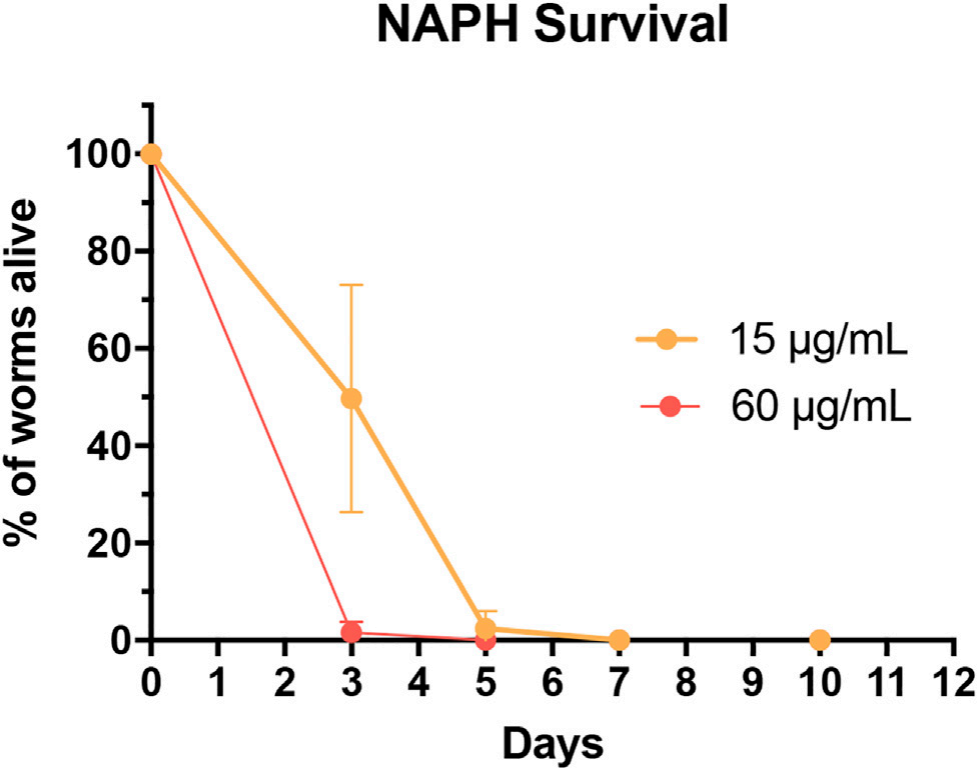
Survival curves of worms treated with 60 μg/ml (red; corresponds to 315.54 µM) and 15 μg/ml (orange; corresponds to 78.89 µM) NAPH. Dots represent counting events as mean of three parallel experiments ±SD. The survival curve for DMSO-treated worms (throughout the experiment at 100%) is not shown.

These findings prompted us to investigate the A/S-mix and eight hydroxynaphthoquinones (most of them being present in the A/S-mix) for their nematocidal effect in *C. elegans*. The nematocidal activity was evaluated by analysis of survival over time. Vehicle-treated (1% DMSO) and compound treated (60 and 15 μg/ml) N2 wild type worm cohorts were compared. After 10 days of treatment a decreased survival rate was confirmed for the A/S-mix, as well as for DMAS and NAPH. On the contrary, all worms treated with the vehicle control (DMSO 1%) survived until day 10 of the experiment (Figure 8B; Table 2). IVS, S, A, S-A (42:58), and DS showed no significant effect on the survival rate of worms (data not shown), whereas NAPH showed the most pronounced nematocidal effect, when tested at 15 and 60 μg/ml, respectively. In Figure 9 the corresponding survival curves are outlined, showing that only 50% of worms survived the first 3 days of treatment, when incubated with 15 μg/ml NAPH. At the third day of treatment, almost 100% of the nematodes were dead in the 60 μg/ml NAPH cohort (Figure 9).

**TABLE 2:** Effect of hydroxynaphthoquinones and samples derived from *Alkanna tinctoria* L. Tausch on *C. elegans* survival and fat accumulation. Survival rates (%) after 10 days upon treatment, as well as fat accumulation assessed as Nile red fluorescence (% of vehicle control). Vehicle control was 1% DMSO in both assays. *N* is the total number of worms assayed for the respective assay. Significance was assessed by one-way ANOVA with Dunnett’s post-test.

Sample	Concentration	Survival assay		Fat accumulation	
		% Survival after 10 days ±SD	*N*	% Fluorescence of vehicle control ±SD	*N*
DMSO	1%	100	97	100	50
A/S-mix	60 μg/ml	53.17 ± 21.68 ***	89	n.d	—
	15 μg/ml	73.93 ± 5.53 *	96	n.d	—
	10 μg/ml	n.d	—	180.2 ± 51.52 *	56
	2.5 μg/ml	n.d	—	174.3 ± 29.0	71
HE	25 μg/ml	n.d	—	175.4 ± 37.5	54
	10 μg/ml	n.d	—	197.8 ± 11.2 *	50
	2.5 μg/ml	n.d	—	160.7 ± 35.2	44
DMAS	60 μg/ml	62.22 ± 17.36 ***	83	n.d	—
	15 μg/ml	73.87 ± 1.61 *	98	n.d	—
NAPH	60 μg/ml	0.00 ± 0.00 ****	92	n.d	—
	15 μg/ml	0.00 ± 0.00 ****	67	n.d	—

*p*-values (*p < 0.05; ***p < 0.001; ****p < 0.0001) were considered as statistically significant.

## 4 Discussion

n the present study, we aimed at investigating the activity of various hydroxynaphthoquinones (A/S and their derivatives) in regard to adipogenesis and their potential impact on nematode fat accumulation and survival. Our ultimate goal was to fill the literature gap and expand the biological properties of A/S and their derivatives for the discovery of potential antidiabetic and anthelmintic drugs. Therefore, we employed two study models, the 3T3-L1 pre-adipocyte cells (*in vitro*) and the *C. elegans* nematode (*in vivo*), and approximated a structure-activity relationships (SAR). The tested compounds were selected from a series of A/S derivatives that share a similar naphthoquinone structure and constitute the main bioactive components of several plants of the Boraginaceae family, such as *Alkanna tinctoria* (L.) Tausch, *L. erythrorhizon* Siebold and Zucc., together with the parent moiety, naphthazarin.

Before assessing the cytotoxicity of the individual A/S compounds, we first tested the cytotoxicity of a hexane extract (HE) from *A. tinctoria* (L.) Tausch roots, and a mixture consisting of A/S derivatives (A/S-mix). Cell viability assays (after 24 h) indicated that A/S-mix was more active than HE, demonstrating an IC_50_ value of 4.21 μM (∼1.51 μg/ml); the respective IC_50_ value of HE was estimated to be more than 2 times higher (3.9 μg/ml). This observation is consistent with the content of HE and A/S-mix in A/S compounds. Specifically, A/S-mix is mainly composed of A/S esters (β,β,-dimethyl-acryl-, isovaleryl- and acetyl-A/S) and A/S, whereas the HE contains more lipophilic bulk compounds, such as lipids, waxes, and polymeric pigments (Papageorgiou and Assimopoulou, 2003). This is in concordance with our results concerning the isolation of A/S-mix from the HE of *A. tinctoria* (L.) Tausch roots; A/S-mix constitutes ∼65 wt% of the HE. The picture was similar for the 48-h cell viability assay, with the A/S-mix being more cytotoxic than HE.

The cytotoxic analysis of A/S and their derivatives suggested that the chemical structures of the tested compounds project a significant role towards their activity in 3T3-L1 cells. This can be clearly visualized in Figure 3, where, for example, the IC_50_ values (after 24 h) of the parent naphthazarin moiety, the two enantiomers (A/S), and the 42:58 mixture of S-A, were >50% lower than those of the respective hydrogen- or acyl-substituted derivatives. These data corroborate previous findings, highlighting the biological importance of dihydroxy substitutions at C-5 and C-8 of the aromatic ring and the free hydroxyl group in the side chain (Ali et al., 2011; Ordoudi et al., 2011; Arampatzis et al., 2021). Comparing the various enantiomeric ratios of A/S, it is apparent that differences in toxicity do exist, though most of them are not significant, except for 2 μM, where A and S-A (42:58) proved significantly less toxic than S at 24 and 48 h. Furthermore, the reduced toxicity of A/S esters may be correlated with the absence of a side-chain hydroxyl group and the concomitant effects on lipophilicity and steric hindrance phenomena. Accordingly, compounds with increased lipophilicity (high Log *p* values) show decreased biological activities, due to strong interactions with the phospholipid bilayer of the cytoplasmic membrane, while bulky and non-planar molecular structures might be associated with steric hindrance phenomena.

After defining the non-toxic concentrations of the hydroxynaphthoquinones, we proceeded to 3T3-L1 cell differentiation by using the most active compounds and in combination with insulin. Previous studies have reported that S and its derivatives (e.g., β-hydroxy-isovaleryl-shikonin) were able to inhibit adipogenesis at varying concentrations, such as 0.5–2 μM (Lee et al., 2010; Gwon et al., 2013; Ha et al., 2016). Our results are consistent with these previous findings, as all tested A/S compounds at 1 μM managed to hinder pre-adipocyte differentiation to different extents (Figure 4). The anti-adipogenic activity was also evident at a lower concentration (100 nM), yet it was not as pronounced as in the case of 1 μM, which suggested the presence of a concentration-dependent mechanism. Furthermore, as it was also observed in the cytotoxicity experiments, there were considerable differences between the various A/S compounds in the adipogenesis study as well. Remarkably, the parent enantiomers were found to be more active than the rest of hydroxynaphthoquinones. This again is in concordance with previous studies, which suggested that the free hydroxyl group in the side chain is important for an enhanced biological activity (Ordoudi et al., 2011).

It has also been proposed that oxidative stress is associated with obesity and fat accumulation in humans and mice, while reactive oxygen species (ROS) production is taking place during *in vitro* adipogenesis in 3T3-L1 cells (Lee H. et al., 2009), acting as an inducer of lipid production. Several studies have demonstrated the potent antioxidant properties of A/S and derivatives, while our group has additionally underscored their ability to act as effective radical scavengers (Assimopoulou et al., 2004; Assimopoulou and Papageorgiou, 2005; Ordoudi et al., 2011). Therefore, we can assume that the inhibitory action of A/S and their derivatives on 3T3-L1 cell differentiation is explained by the interaction of these compounds with ROS.

In an attempt to explore whether A/S and their derivatives possess insulin-like properties, insulin was completely replaced by the hydroxynaphthoquinones in the induction medium, according to similar studies (Tsave et al., 2016; Hasan et al., 2017; Tsave et al., 2018). Our results showed that A/S compounds led to a concentration-dependent attenuation of adipogenesis, hence no insulin-like activity was observed. However, once more we were able to detect differences between the observed activities of the various A/S samples; the two enantiomers together with DS (at 1 μM) showed the strongest activity.

To further evaluate the effect of A/S and their derivatives on adipogenesis and to get a deeper insight at the molecular level, we analyzed the relative mRNA expression of adipogenesis-specific genes. PPAR-γ is a transcriptional factor that predominates the early stages of adipogenesis and determines its progression. Upon its activation, PPAR-γ induces the expression of a large group of genes that generate the adipocyte phenotype (Tang and Lane, 2012). All tested compounds impeded the expression of PPAR-γ, yet S, A, and S-A (42:58) demonstrated the lowest fold increases, thus validating the findings from the ORO assay. This observation is in line with literature, where S significantly inhibited the expression of PPAR-γ at concentrations from 0.5–2 μM (Lee et al., 2010; Gwon et al., 2013). Moreover, we were interested to assess the expression of PPAR-γ in samples treated only with the selected hydroxynaphthoquinones. The resulting data were in line with those obtained from the ORO assay; no compound exhibited an insulin-like activity. Nevertheless, DMAS showed the highest fold increase.

Besides PPAR-γ, we were prompted to examine the expression of ADIPOQ; an adipokine that is dramatically induced during adipogenesis and especially in mature adipocytes. Based on the results, it was evident that S and A were the most active compounds, when administered together with insulin causing the strongest suppression in ADIPOQ expression among all tested compounds. In general, RT-PCR analysis and data from ORO assay showed a similar pattern as is evidenced by comparing Figures 4, 7. For example, A inhibited almost to 100% the formation of lipids (Figure 4), which was translated to a null ADIPOQ expression. Furthermore, Lee and co-workers showed that S could strongly inhibit ADIPOQ expression at 2 μM (Lee H.-Y. et al., 2009). Our findings for S were similar, since we observed that S could reduce the expression of ADIPOQ at 1 μM. Concerning cell differentiation in the absence of insulin, all samples exhibited weakened fold increases too. Interestingly, DS showed complete impediment of ADIPOQ expression, followed by the rest of compounds, which yet demonstrated smaller fold increases than the positive control sample. Overall, as in the ORO assay, no insulin-like activity was observed for any of the hydroxynaphthoquinones. Taken together, the case of the herein selected compounds in fat accumulation formulates a representative approach, considering distinct structural and biochemical characteristics that might serve in meaningful comparisons emphasizing the impact of structural selectivity in biomimesis.

Concerning the *in vivo* experiments, results from the *C. elegans* fat accumulation assay indicated that low concentrations of HE and A/S-mix increased worm fluorescence (lipid accumulation), whereas higher concentrations of particularly the A/S-mix led to a significant nematotoxicity; hence, implying an anthelmintic activity. Consequently, we tested the A/S-mix and a series of pure A/S compounds in a *C. elegans* assay. The *C. elegans* findings revealed a similar pattern with the 3T3-L1 cytotoxicity data: The hydrophilic parent compound NAPH with a high relative topological polar surface area, due to lacking the side chain and possessing a planar structure, showed increased toxicity compared to the A/S esters (e.g., DMAS or IVS). The anthelmintic activity of naphthoquinones further showed an inverse correlation with the molecular eccentricity and SP3 character. However, our data propose that since naphthazarin is the only highly active molecule, further experiments with naphthazarin analogues are necessary to complement a more robust SAR. Differences between the *C. elegans* assays and *in vitro* cultured cells should be considered to possibly affect experimental outcomes. Compounds are more likely to enter *in vitro* cultured cells than into *C. elegans*, which is protected by a poorly permeable cuticle. Further, bacteria and intact animals also possess a wide range of metabolizing enzymes and excreting mechanisms (Burns et al., 2010). In regard to NAPH toxicity, a previous publication has reported to extend the lifespan of *C. elegans* when worms were treated with different concentrations of NAPH, ranging from 100 to 500 μM (Hunt et al., 2011). However, it is noteworthy that the higher concentrations of NAPH used in the abovementioned publication were applied to solid agar medium, while in the present work NAPH was tested in a 96-well plate format in liquid medium; it has been reported that enhanced bioavailability can be achieved in liquid medium (Zheng et al., 2013). Molecular targets through which the anthelmintic activity of NAPH is exerted have not been determined in this study. Different approaches to achieve this have been recently presented, e.g., (Burns et al., 2015).

Overall, the present work aimed to approach a SAR study to explore the effect of different A/S derivatives on two study models, *in vitro* and *in vivo*. The reduced lipid accumulation we observed in 3T3-L1 cells was more pronounced for the samples of A and S, probably attributed to the presence of the free hydroxyl group. Contrariwise, fat accumulation in the worm model seemed to be promoted in comparison with the control sample; as evidenced by an increased Nile red fluorescence of worms at lower concentrations of A/S-mix, while higher concentrations were too toxic for evaluation. This indicated that the nematocidal activity of A/S compounds might have prevailed over the potential *in vivo* anti-adipogenic effect. A significant nematocidal activity of A/S derivatives was later confirmed for DMAS and NAPH. This has been the first time that *A. tinctoria* (L.) Tausch naphthoquinones were tested on *C. elegans*, while—to the best of our knowledge—their use as a natural herbal remedy with nematocidal activity has been demonstrated for the first time. Both *in vitro* cell culture models as well as *in vivo* screenings using the small organism *C. elegans* are valuable tools to initiate or refine the conceptual basis of naphthoquinone actions as anti-obesity/antidiabetic agents. However, it is beyond question that more detailed analyses in higher organisms are needed. The presented findings provide a solid basis for further investigations crucial for both the discovery of new antidiabetic and anthelmintic drugs to combat nematode infections.

## Data Availability

The original contributions presented in the study are included in the article/supplementary material, further inquiries can be directed to the corresponding author.

## Abbreviations

A/S, Alkannin/Shikonin; A/S-mix, A/S mixture; ACS, Acetyl-shikonin; ADIPOQ, Adiponectin; C/EBPα, CCAAT/enhancer binding protein α; DM, Diabetes mellitus; DMAS, β,β-Dimethyl-acryl-shikonin; DS, Deoxy-shikonin; ERK1/2, Extracellular signal-regulated kinases 1 and 2; FFA4, Free fatty acid receptor 4; FKBP1B, FK506 binding protein 1B; HE, Hexane extract; IVS, Isovaleryl-shikonin; KLF15, Kruppel-like factor 15; NAPH, Naphthazarin; NGM, Nematode growth medium; ORO, Oil red O; PPAR-γ, Peroxisome proliferator-activated receptor γ; PS, Propionyl shikonin; PTP1B, Protein tyrosine phosphate 1B; SAR, Structure activity relationship; SREBP1C, Sterol regulatory element binding protein 1C.
